# ﻿Evaluation of genetic diversity and population structure in *Leptobotiamicrophthalma* Fu & Ye, 1983 (Cypriniformes, Cobitidae)

**DOI:** 10.3897/zookeys.1121.85953

**Published:** 2022-09-12

**Authors:** Dongqi Liu, Shiming Zhang, Xinyu Zuo, Yi Zheng, Jing Li

**Affiliations:** 1 Sichuan Province Key Laboratory of Characteristic Biological Resources of Dry and Hot River Valley, School of Biological and Chemical Engineering, Panzhihua University, Panzhihua, 617000, China Panzhihua University Panzhihua China; 2 Upper Changjiang River Bureau of Hydrological and Water Resources Survey, Chongqing, 400000, China Upper Changjiang River Bureau of Hydrological and Water Resources Survey Chongqing China

**Keywords:** Conservation biology, fish ecology, microsatellites, restoration

## Abstract

This paper reports the first account about dynamic changes on genetic diversity and population structure of *Leptobotiamicrophthalma* in the Yangtze River drainage due to dam constructions. The genetic diversity and population structure of twelve populations of *L.microphthalma* collected in 2010 and 2020 were estimated using 12 nuclear microsatellite markers. Reduction of genetic diversity between 2010 and 2020 was not significant in a paired *t*-test (*p* > 0.05), but population structure of *L.microphthalma* had a tendency to change: the genetic differentiation (*F*st) among the five populations collected in 2010 were all insignificant (*p* > 0.05). However, differentiation (*F*st) among some populations collected in 2020 were significant (*p* < 0.05), which indicated the population structure of *L.microphthalma* was changing. Correlation analysis indicated that negative correlations between the genetic diversities and geographical elevations among populations were significant for seven populations collected in 2020 (r = -0.819, *p* = 0.039), which means that populations of *L.microphthalma* in high elevation regions were more vulnerable than those in low elevation regions. Finally, some suggestions for conservation and restoration are proposed, such as artificial propagation, to prevent the further reduction of genetic diversity and population resources.

## ﻿Introduction

*Leptobotiamicrophthalma* Fu & Ye, 1983 (Cypriniformes: Cobitidae) is an important benthic commercial fish with high ornamental and edible value. It is a unique Chinese species (Ding 1994) and endemic to the middle and upper reaches of the Yangtze River and its tributaries. It is known in China and international markets for its vivid color (Cao et al. 2007). In recent years, *L.microphthalma* populations have declined greatly due to dam construction, over-fishing, and environmental deterioration, including the destruction of prey and breeding grounds (Yue and Chen 1998). The annual catch was 2000 kg prior to the 2000s, but has decreased to no more than 500 kg per year in recent years (Shen et al. 2017).

The species is often found near gravel and rock crevice habitats on the bottom of rivers and streams with swift currents (Ding 1994). This species often goes up-stream to spawn, and spawning occurs from April to June, with eggs and developing larvae drifting with the currents (Liang et al. 2009). Thus, the development of embryos and the growth of larvae requires relatively long and continuous rivers. However, this natural requirement conflicts with the construction of hydropower stations on the Yangtze River (Yue and Chen 1998). With the construction of Three Gorges Dam and other cascade hydropower stations within its distribution, *L.microphthalma* is suffering from severe threats to its survival (Liu et al. 2014a). Thus, the genetic connection and communication between populations may be interrupted, resulting in bottlenecks and inbreeding. As a result, the genetic diversities may decrease. If wild species are to survive environmental changes beyond the limits of developmental plasticity, they must have an available and viable genetic diversity pool. If not, extinction would appear to be inevitable (Frankel 1983). Today, artificial propagation and breeding techniques are successful (Ku et al. 1999), and the release of cultured juveniles into rivers has been proposed as one conservation strategy, and has been carried out for several years on a small scale in the upper reaches of the Yangtze River (Zhou et al. 2007). These releasing programs can increase the quantity, but they may have influences on genetic diversities and structures of wild populations of *L.microphthalma*.

Considering the present and potential environmental threats from artificial negative factors to *L.microphthalma* populations and the influence of releasing program, it is necessary to monitor the dynamic population genetic status of *L.microphthalma*. Shen et al. (2017) studied the diversity of two populations (only 108 samples) using mtDNA control region. However, the comparative study of *L.microphthalma* during different times using different genetic markers in a larger quantity of samples has not been made to date. In the present study, twelve microsatellites were used to assess genetic diversity and population structure of twelve *L.microphthalma* populations including 280 individuals collected in upper and middle drainages of the Yangtze River from 2010 to 2020. The genetic diversity and population structure of *L.microphthalma* in 2010 was then compared with that in 2020.

In this study, the population genetic diversity of *L.microphthalma* in the upper reaches of the Yangtze River was studied to understand the genetic background of *L.microphthalma*, and to provide basic knowledge for germplasm protection of *L.microphthalma*. At the same time, it provides a scientific basis for evaluating and predicting the impact of cascade power station development on aquatic animals and ecological environments of the Yangtze River.

## ﻿Materials and methods

### ﻿Sample collection and microsatellites amplification

**Figure 1.**
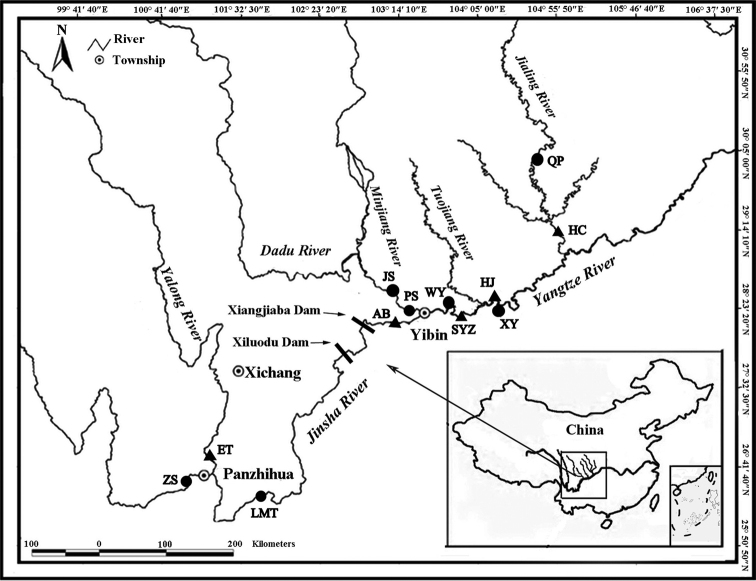
Sampling localities of China (black triangles indicate collection in 2010; black circles indicate collection in 2020) of *L.microphthalma*. For full names of population codes, see Table 1.

Fins from 280 individuals of *L.microphthalma* were collected from the middle and upper Yangtze River drainage (Table 1). In fact, our sampling covered most of the distribution area of the species. Five populations of *L.microphthalma* were collected from the Ertan part of the Yalong River (**ET**), the Anbian part of the Jinsha River (**AB**), the Shiyuanzi part of the Yangtze River (**SYZ**), the Hejiang part of the Yangtze River (**HJ**), and the Hechuan part of the Jialing River (**HC**) in 2010. Seven populations of *L.microphthalma* were collected from the Zhuangshang part of the Jinsha River (**ZS**), the Laomatian part of the Jinsha River (**LMT**), the Juexi part of the Min River (**JX**), the Pingshan part of the Yangtze River (**PS**), the Wayao part of the Yangtze River (**WY**), the Xuyong part of the Yangtze River (**XY**), and the Qiping part of the Jialing River (**QP**) in 2020 (Fig. 1). In order to reduce experimental errors caused by different fish ages, 247 individuals, aged between 3 and 4 years old, were selected from 280 samples, according to the relationship between age and body length (Cao et al. 2007).

**Table 1.** Sample information of *L.microphthalma*.

Population code	Population name	River	Sample size		Elevation (m)	Sampling time
			N_1_	N_2_		
HJ	Hejiang	Yangtze River	15	13	235	Apr 2010
HC	Hechuan	Jialing River	21	19	200	Jul 2010
ET	Ertan	Yalong River	22	17	991	Aug 2010
AB	Anbian	Jinsha River	24	23	421	May 2010
SYZ	Shiyuanzi	Yangtze River	22	21	281	Jun 2010
WY	Wayao	Yangtze River	24	22	272	Aug 2020
XY	Xuyong	Yangtze River	21	18	235	Sep 2020
JS	Juexi	Minjiang River	25	22	311	Nov 2020
QP	Qingping	Jialing River	28	23	284	Oct 2020
PS	Pingshan	Minjiang River	24	23	273	May 2020
ZS	Zhuangshang	Jinsha River	26	22	1103	May 2020
LMT	Laomatian	Jinsha River	28	24	890	Nov 2020
Total			280	247		

N_1_, Number of samples collected; N_2_, Number of samples used for analyses.

Twelve microsatellite loci (XY12, XY13, XY14, XY21, XY27, XY32, XY35, XY37, XY38, XY41, XY43, and XY45) specifically developed for *L.microphthalma* (Liu et al. 2014a, b) were used as described in Liu et al. (2018). Procedures of polymerase chain reaction (PCR) amplification, electrophoresis, and fluorescent microsatellites were speciﬁcally developed in Liu et al. (2020) and the methodology was followed herein.

### ﻿Genetic diversity and landscape analysis

The software GenAlEx 6.501 was used to statistically analyze observed heterozygosity (***H*o**), expected heterozygosity (***H*e**), and polymorphism information content (**PIC**) (Peakall and Smouse 2006). Average allelic richness by population (***A*r**) and private allelic richness (***PA*r**) across 12 nuclear loci were calculated using HP-RARE 2.2 (Kalinowski 2005), which uses the rarefaction procedure to account for variable sample sizes. Paired *t*-test was used to compare *A*r and *PA*r. The correlation between genetic diversity and elevation was calculated.

The software POPGENE 2.4 was used to calculate Hardy–Weinberg equilibrium (HWE) and pairwise genetic differentiation (***F*st**) (Francis et al. 1999). A Bayesian approach was applied to explore the population genetic structure using STRUCTURE 2.2.1 (Pritchard et al. 2000). The number of groups K was set to 1–9, and each K was repeated five times. The calculation results were packaged, and the optimal K value was analyzed by STRUCTURE Harvester. As a best choice to maximize ΔK corresponding to the K value (including ΔK = mean|(L ‘(K) | / sd (K) (L), L’ (K) as the likelihood distribution rate averages, |’L’ (K)| for the second round of likelihood distribution rate the absolute value of the mean), Repeated sampling analysis is conducted on the results of structure operation to obtain the Q value corresponding to the optimal K value, and the structure graph is drawn according to the Q value gene flow and landscape analyses (Evanno et al. 2005). Population structure was evaluated using the analysis of molecular variance model (AMOVA) in the ARLEQUIN 2.0 (Excoffier et al. 1992).

The BOTTLENECK tests for the departure from mutation drift equilibrium based on heterozygosity, excess or deficiency. The bottleneck compares heterozygosity expected (*H*e) at Hardy-Weinberg equilibrium to the heterozygosity expected (***H*eq**) at mutation drift equilibrium in the same sample. All the three models of mutation were used to calculate *H*eq: the strict one stepwise mutation model (**SMM**; Kimura and Ohta 1978), the infinite allele model (**IAM**; Kimura and Crow 1964) and two-phase model (**TPM**; Di Rienzo et al. 1994). FSTAT 1.8.2 was used to estimate inbreeding coefficient (***F*is**).

## ﻿Results

### ﻿Genetic data of diversities

The allelic richness (*A*r) of populations ranged from 11.17 in XY to 15.36 in QP (Table 2). Average observed heterozygosity (*H*o) varied from 0.753 in JX to 0.899 in XY. Average expected heterozygosity (*H*e) varied from 0.839 in XY to 0.869 in PS. Most average values of *H*o were lower than those of expected heterozygosity (*H*e) in all populations except for ET, LMT, XY, and QP (Table 2). Average polymorphic information content (*PIC*) varied from 0.785 in ZS to 0.852 in HC (Table 2). The private allelic richness (*PA*r) varied from 1.63 in ET to 21.36 in LMT, and LMT also showed the highest number of private alleles, while the lowest number of private alleles was found in ET (Table 2). All differences of genetic diversity (*PIC* and *A*r) between the 12 populations were insignificant in paired *t*-tests (*p* > 0.05). Significant negative correlations between the genetic variability and elevations among populations were found for the seven populations in 2020 (r = -0.819, *p* = 0.039), but there were no significant correlations for the five populations in 2010.

**Table 2.** Genetic variability of *L.microphthalma* populations. For full names of population codes, see Table 1.

Population	*H*o	*H*e	*PIC*	*A*r	*PA*r	*F*is
HJ	0.838	0.849	0.844	13.25	6.45	0.134
HC	0.798	0.853	0.852	12.14	3.52	0.244
ET	0.878	0.846	0.808	13.72	1.63	0.102
AB	0.808	0.866	0.828	14.51	15.96	0.100
SYZ	0.793	0.862	0.838	14.62	7.01	0.217
WY	0.755	0.841	0.836	14.32	6.57	0.318
XY	0.899	0.839	0.842	11.17	8.13	0.198
JS	0.753	0.848	0.802	14.44	9.05	0.218
QP	0.863	0.846	0.814	15.36	8.9	0.651
PS	0.862	0.869	0.825	14.14	5.48	0.365
ZS	0.828	0.840	0.785	13.45	13.9	0.307
LMT	0.873	0.856	0.798	14.17	21.36	0.747

Ho, observed heterozygosity; He, expected heterozygosity; PIC, polymorphism information content; Ar, allelic richness; PAr, private allelic richness; Fis, inbreeding coefficients.

### ﻿Population genetic demography and structure analyses

The 12 populations were divided into three groups based on sampling locations: upper group (ET, ZS, and LMT), middle group (HJ, SYZ, AB, XY, JX, PS, and WY), and lower group (HC and QP). An AMOVA performed in five populations (SYZ, ET, HC, AB, and HJ) sampled in 2010 indicated molecular variance between groups, between populations, and within populations were all insignificant (*p* > 0.05). Although AMOVA in seven populations (XY, WY, QP, PS, LMT, ZS, and JX) sampled in 2020 indicated that molecular diversity between groups and between populations within sites were insignificant, variance within populations (98.16%) was significant (*p* < 0.05).

**Figure 2.**
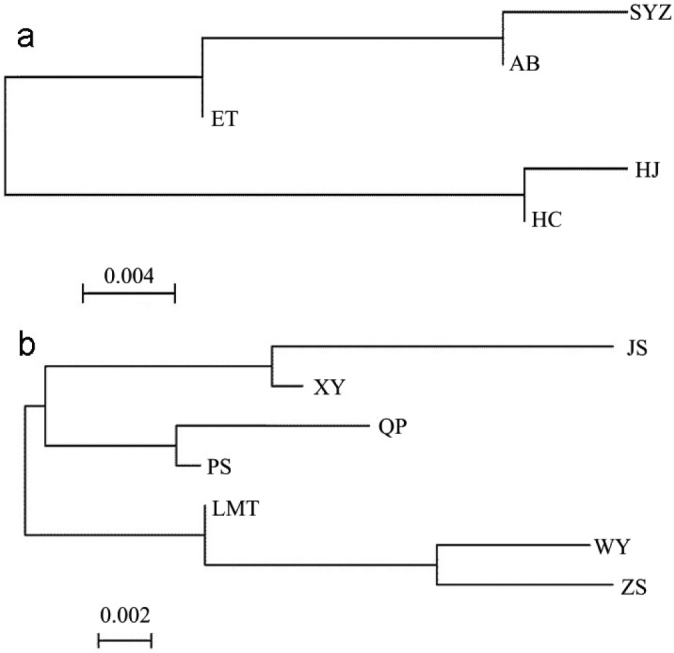
Neighbor-joining tree based on *F*st. For full names of population codes, see Table 1.

Pairwise *F*st between populations varied from 0.022 to 0.330 (Table 3). *F*st between five populations sampled in 2010 (HJ, HC, ET, AB, and SYZ) were all insignificant (*p* > 0.05; Table 3). However, some *F*st between seven populations sampled in 2020 (JX, WY, XY, LMT, QP, PS, and ZS) were significant (*p* < 0.05), including *F*st between QP and ZS, and LMT and JX (Table 3). Some *F*st between five populations (2010) and seven populations (2020) were significant (*p* < 0.05) including *F*st between ZS and SYZ, and between LMT and HC (Table 3). Two neighbor-joining trees were built based on *F*st values among five populations (2010) and among seven populations (2020), respectively. HC and HJ populations were clustered together and the AB, SYZ, and ET populations were clustered together in the neighbor-joining tree (Fig. 2a). JX and XY, and QP and PS were clustered together in the neighbor-joining tree, respectively (Fig. 2b). LMT, WY, and ZS populations were also clustered together in the neighbor-joining tree (Fig. 2b).

**Table 3.** Pairwise genetic differentiation of *L.microphthalma* populations. For full names of population codes, see Table 1.

	HJ	HC	ET	AB	SYZ	WY	XY	JS	QP	PS	ZS
HC	0.037										
ET	0.079	0.063									
AB	0.046	0.037	0.068								
SYZ	0.075	0.022	0.048	0.073							
WY	0.048	0.023	0.062	0.065	0.066						
XY	0.078	0.048	0.077	0.078	0.047	0.078					
JS	0.074	0.056	0.079	0.057	0.050	0.089	0.056				
QP	0.032	0.053	0.083	0.056	0.080	0.058	0.078	0.047			
PS	0.047	0.067	0.050	0.040	0.058	0.057	0.054	0.064	0.064		
ZS	0.074	0.073	0.053	0.083	0.270*	0.060	0.064	0.058	0.330*	0.052	
LMT	0.069	0.160*	0.084	0.079	0.087	0.073	0.078	0.214*	0.080	0.073	0.062

* *P* < 0.05.

**Figure 3.**
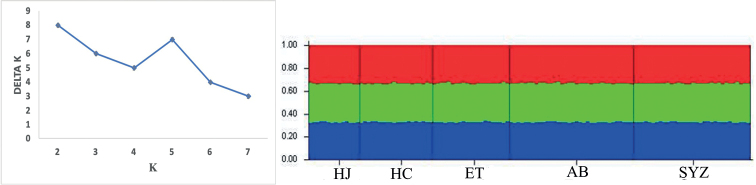
Structure results and maximum DK values of the *L.microphthalma* populations collected in 2010. For full names of population codes, see Table 1.

**Figure 4.**
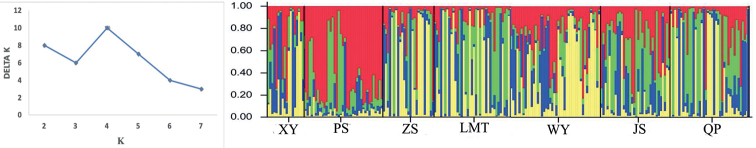
Structure results and maximum DK values of the *L.microphthalma* populations collected in 2020. For full names of population codes, see Table 1.

**Figure 5.**
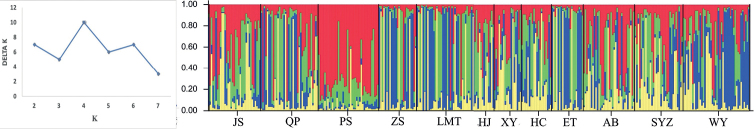
Combined structure results and maximum DK values of the *L.microphthalma* populations collected in 2010 and 2020. For full names of population codes, see Table 1.

STRUCTURE analysis was applied in five populations (2010), seven populations (2020), and all twelve populations. The optimal K value in five populations (2010) was 3. However, the five populations did not form independent clusters for K = 3, with each sample in effect having equal probability of belonging to any of those clusters in either analysis (Fig. 3). The ΔK statistic estimated the best supported number of a posterior genetic clusters at K = 4 in either seven populations (Fig. 4; 2020) or all twelve populations (Fig. 5). There was no apparent clustering of individuals into groups for K = 4 in seven populations (Fig. 4; 2020) or all twelve populations (Fig. 5), but there was a more-or-less unequal probability of every individual belonging to each cluster. Individuals in each population are becoming divergent to each other, which is consistent with the AMOVA analysis. As such, there was a trend of overall genetic clustering. Analysis using the coalescent-based method based on the IM model showed that the level of gene flow among populations was very limited: there was no significant gene flow in both five populations (2010) and seven populations (2020).

The results of the bottlenecks are summarized in Table 4 and show every population tested under three possible mutation models. The probability values of one-tailed Wilcoxon test for heterozygosity excess (*H*e) were < 0.05 in ET, JX, QP, and LMT in the infinite allele model (IAM). Nevertheless, under the stepwise mutation model (SMM) and the two-phase model (TPM), the excess heterozygosity of each population is not significant, and there has been no bottleneck effect or foundation effect in the past (Table 4). The inbreeding coefficient (*F*is) of the 12 populations ranged from 0.100 in AB to 0.747 in ZS (Table 2). The inbreeding coefficient (*F*is) of LMT and QP were significant (*P* < 0.05), while that of the other populations were not significant.

**Table 4.** Probabilities of Wilcoxon test of *L.microphthalma* populations for mutation drift equilibrium (bottleneck) under three mutation models. For full names of population codes, see Table 1.

Population	I.A.M	S.M.M	T.P.M
HJ	0.1354	0.5542	0.3441
HC	0.0676	0.6883	0.7981
ET	0.0436*	0.7449	0.7448
AB	0.2358	0.2778	0.3509
SYZ	0.1557	0.9807	0.3127
XY	0.1829	0.1129	0.2030
WY	0.0659	0.3367	0.1873
JS	0.0359*	0.0514	0.0859
QP	0.0358*	0.0523	0.0750
PS	0.1235	0.2147	0.1209
ZS	0.0553	0.2445	0.0647
LMT	0.0485*	0.0559	0.0736

* *P* < 0.05 (rejection of mutation drift equilibrium).

## ﻿Discussion

### ﻿Tendency of population genetic variabilities

The important indexes of population genetic diversity are heterozygosity and PIC. The higher the heterozygosity and PIC are, the greater the genetic variation; the higher the genetic diversity leads to greater stability of the population. Negative correlations among the genetic variabilities and geographical elevations between populations were significant for seven populations (2020), which indicates that rising elevation is always accompanied by reducing genetic variabilities. Therefore, populations of *L.microphthalma* in upper streams of the Yangtze (higher elevation area) are more fragile than those present downstream (lower elevation) flows. Hence, populations of *L.microphthalma* in upstream Yangtze River and its tributaries are extremely important in conservation and can serve as models for monitoring biodiversity in regions impacted by anthropogenic disturbances.

All populations indicated heterozygosity deficiencies except ET, LMT, XY, and QP. Two previous studies had examined how levels of heterozygosity varied during the course of well-documented demographic challenges. Ruzich et al. (2019) found that heterozygote go up during population reduces in six fish species, and Valsecchi et al. (2004) found that Mediterranean striped dolphins dying early in an epizootic environment were significantly less heterozygous than those dying later. These studies imply that natural selection may sometimes remove relatively homozygous individuals from populations during demographic declines, raising the counter-intuitive possibility that declining populations may in fact be more heterozygous than stable ones. Therefore, four populations (ET, LMT, XY and QP) may have experienced recession recently. Furthermore, the purging of genetic load during population bottlenecks could generate a scenario where relatively homozygous populations do better when faced with a challenge (Haxton et al. 2015).

### ﻿Dynamics of population structure and landscape analysis

The AMOVA analysis indicated that five populations (2010) of *L.microphthalma* exhibited limited genetic differentiation between groups and between populations. *F*st analysis indicated no significant population structure among the sampling locations. Also, the five populations did not form independent clusters in the population structure, with each sample in effect having the same possibility of belonging to any of those clusters in either analysis. There was significant correlation between the observed genetic differentiations and geographical distances in the five populations. This species spawn eggs which usually drift with flood currents downstream, and the adult fish usually swim upstream when the river are flooded (Li et al. 2015). Those life histories traits could accelerate genetic exchanges among groups across their distribution. In the Yangtze River region, the genetic differentiations between the populations of *Coreiusguichenoti* (Sauvage & Dabry, 1874) is also not obvious, which is also due to the movement of floating larvae and eggs (Liu et al. 2021). This is not different to the loss of population genetic structure of *L.microphthalma* due to similarity in breeding and life cycle characteristics, which may also be connected with the indeterminacy of countercurrent swimming distance.

However, there were obvious differences in genetic structure between the seven populations in 2020: *F*st indicated significant differences among some populations. There are no independent components in the structure of the seven populations, but the components of each population were not the same as the others. Although AMOVA indicated that genetic divergences among groups were finite, the genetic divergences that occurred between individuals in a population were significant (*p* < 0.05). Significant differentiation in all populations is not yet formed, but individuals are becoming different to each other within each population. The dams limit genetic connections between upstream and downstream populations of *L.microphthalma* and divide it into smaller independent populations. A robust barrier to dispersal likely exists to restrict genetic exchange among sample locations. The habitats of *L.microphthalma* will become isolated, which influences not only the breeding and development environments of *L.microphthalma*, but also prevents upstream and downstream gene flow. Therefore, there was no inapparent correlation between genetic differences and geographical distance in these seven populations (2020, *p* > 0.05). The individuals of populations are becoming different from each other because of a changing aquatic environment. Therefore, strong structures of populations might be formed in the future. This was evidenced by bottleneck analysis: microsatellite data indicated that some populations collected in 2020 suffered from bottleneck or founder effects under two models (IAM, SMM), which was consistent with *F*is analysis. Also, the construction of the reservoir upstream of the Yangtze River vastly altered the aquatic environments, and might destroy the habitats and breeding areas of *L.microphthalma*. As a consequence of the lack of flowing water, juvenile fish may grow unsuccessfully; thus, many wild populations and their genetic variability will necessarily reduce.

### ﻿Conservation and restoration guidance

In order to protect and restore the germplasm resources of *L.microphthalma*, the fishing of wild parents and back-up parents should be strictly controlled while protecting the spawning grounds and improving the water environmental conditions, so as to maintain the self-healing potential of natural water resources of *L.microphthalma*. Although relevant departments have long established breeding farms of *L.microphthalma*, the numbers of wild parents in the Yangtze River have decreased sharply in recent years, having died in fishing and transportation, which inevitably leads to the shortage of original parents. Seed farms usually breed F1 generations as back-up parents (Liu et al. 2014b). Due to the high cost, the long cycle (2–3 years), and limited seedling cultivation scale of F1 generation back-up parents in the seed farm, the supply of back-up parents in the seed farm is limited and the price is high. Some farms, in order to save costs, often introduce a small amount of seed stock station back-up parents or fry from numerous progenies reserved for parents, and breed on a smaller scale (Liu et al. 2015); hence, the population is not big enough and it is easy to observe the bottleneck effect and inbreeding depression phenomena, which may also cause farms to produce parent group alleles lacking one of the important reasons.

At present, the proliferation and release of seedlings generally takes the form of bidding for government grants. Therefore, in order to better protect and utilize the germplasm resources of *L.microphthalma*, it is suggested that the relevant functional departments should strictly examine the qualification, breeding scale, and parental source of the nursery before release, and strive to make the source of the nursery traceable and of high quality. The genetic background of the released population must be evaluated before release to ensure the stability of the genetic structure of the natural population. Sampling investigations and supervision of the specification, health status, and germplasm status of the released seedlings should be strengthened at the site of release. The growth, biology, genetic diversity, and genetic structure of natural populations in natural water bodies should be monitored regularly after discharge, and the discharge plan should be adjusted according to the monitoring results.
